# Biodiversity of mycobial communities in health and onychomycosis

**DOI:** 10.1038/s41598-022-13074-8

**Published:** 2022-05-25

**Authors:** Michael Olbrich, Anna Lara Ernst, Foteini Beltsiou, Katja Bieber, Sascha Ständer, Melanie Harder, Waltraud Anemüller, Birgit Köhler, Detlef Zillikens, Hauke Busch, Axel Künstner, Ralf J. Ludwig

**Affiliations:** 1 Lübeck Institute for Experimental Dermatology, Institute for Cardiogenetics, University of Lübeck, Ratzeburger Allee 160, 23562 Lübeck, Germany; 2 Institute for Cardiogenetics, University of Lübeck, Lübeck, Germany; 3 Department of Dermatology, Allergy and Venerology, University of Lübeck, Lübeck, Germany; 4 EUROIMMUN AG, Lübeck, Germany

**Keywords:** Microbiome, Fungal infection

## Abstract

Onychomycosis (OM) is a common fungal nail infection. Based on the rich mycobial diversity in healthy toenails, we speculated that this is lost in OM due to the predominance of a single pathogen. We used next generation sequencing to obtain insights into the biodiversity of fungal communities in both healthy individuals and OM patients. By sequencing, a total of 338 operational-taxonomic units were found in OM patients and healthy controls. Interestingly, a classifier distinguished three distinct subsets: healthy controls and two groups within OM patients with either a low or high abundance of *Trichophyton*. Diversity per sample was decreased in controls compared to cases with low *Trichophyton* abundance (LTA), while cases with a high *Trichophyton* abundance (HTA) showed a lower diversity. Variation of mycobial communities between the samples showed shifts in the community structure between cases and controls—mainly driven by HTA cases. Indeed, LTA cases had a fungal β-diversity undistinguishable from that of healthy controls. Collectively, our data provides an in-depth characterization of fungal diversity in health and OM. Our findings also suggest that onychomycosis develops either through pathogen-driven mechanisms, i.e., in HTA cases, or through host and/or environmental factors, i.e., in cases with a low *Trichophyton* abundance.

## Introduction

Onychomycosis (OM) is a fungal infection of the nail that accounts for around 50% of all worldwide nail disorders^1,2^. The infection occurs in about 10% of the adult population with a higher prevalence in males and elderly people^2–4^. Patients exhibit symptoms such as white or yellow nail discoloration, thickening, roughness, and separation of the nail from the nail bed. The current understanding of OM, as a (mostly) single pathogen-driven infection is based on its current clinical diagnostics, specifically microscopy and culture of clinical samples.

By contrast, multiplex PCR, a recently established diagnostic tool for fungal skin infections, showed a presence of more than one fungal pathogens in up to 32% of the investigated nail samples in OM patients^5,6^. However, multiplex systems are a targeted approach that is designed to detect fungal DNA using species-specific primers^7^. Hence, they are limited to the detection of species that are targeted in the respective assay. By contrast, next-generation sequencing (NGS) yields large numbers of reads and can provide additional, profound insights into the fungal diversity of OM, as it facilitates detection of dominant communities as well as low-abundant and rare taxa^8–11^. In an effort to map the fungal diversity of 14 anatomical sites in clinically healthy individuals, it was found that the toenail fungal community was largely dominated by *Malassezia* at the genus level^12^.

Thus, we hypothesized that *Malassezia* might have a protective effect against other fungal skin pathogens by occupation of the niche. In addition, predicted on the data from multiplex PCR that detects more than one pathogen in OM, we assumed that the composition of pathogens in OM is even more diverse. To challenge these assumptions, we applied internal transcribed spacer 2 (ITS2) sequencing to toenail samples of confirmed OM cases to quantify the fungal microbiome (mycobiome) in human OM. We further included nail specimen from clinically healthy nails, in which neither microscopy, culture nor multiplex PCR detected any fungal pathogen. This identified 338 fungal operational-taxonomic units (OTUs) in all samples. Overall, the results provide detailed insights into the composition of fungal communities in toenails, suggesting two distinct pathogenic pathways in OM pathogenesis.

## Materials and methods

### Patient and sample collection

**Figure 1:**
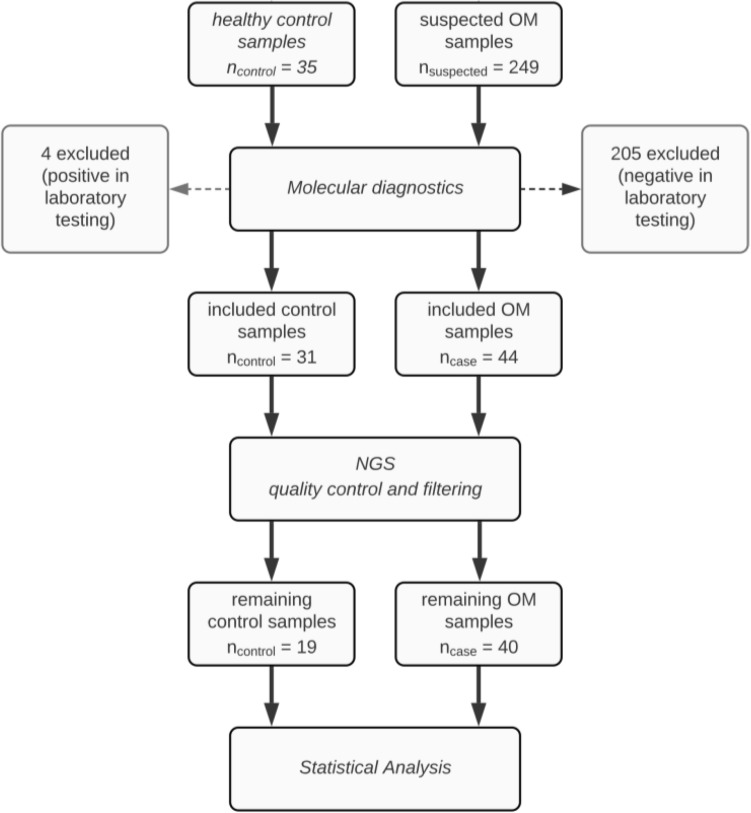
Flowchart of cohort collocation.

Samples were collected from patients treated the in- and out-patient clinic of the Department of Dermatology at the UKSH in Lübeck in the form of toenail scrapings^13^. Patients with diseases that are associated with nail changes, such as psoriasis, alopecia areata or others were excluded. Sample material was obtained from a total of 249 patients with suspected OM. In addition, 35 healthy controls with no clinical evidence of OM were recruited. All patients and controls underwent routine and molecular OM laboratory testing. Of the 249 patients with suspected OM, the diagnosis was confirmed in 44 cases. Thus 205/249 patients with inconclusive results were excluded. Among the 35 healthy controls, routine and/or molecular OM diagnosis showed positive results. Thus, 4/35 controls were excluded and 31 healthy controls were included in the analysis. Quality control procedure post sequencing resulted in a total of 59 remaining samples, of which 40 were confirmed OM cases (25 males, 15 females, average age 61.3 ± 12.5) and 19 healthy controls (6 males, 13 females, average age 42.7 ± 16.3) (Fig. 1, Table 1). All experiments with human samples were performed after written informed consent had been obtained and were approved by the ethical committee of the Medical Faculty of the University of Lübeck (#17-066) in accordance with the Declaration of Helsinki.

**Table 1:** Covariate data.

Group	Sex (f|m)	Age (mean|sd)	Pets^1^ (no|yes)	Therapy^2^ (no|yes)
Control	13|6	42.74|16.26	15|4	19|0
**Case**	15|25	61.33|12.47	29|11	33|7
Case LTA	12|12	60.17|12.71	18|6	21|3
Case HTA	3|13	63.06|12.29	11|5	12|4

List of covariates grouped by case and control samples as well as the identified classification into low *Trichophyton* abundance (LTA) and high *Trichophyton* abundance (HTA) subgroups ^1^indicates if pets are in the household and/or the individual has contact to animals on a regular basis ^2^indicates if the person had applied any treatment (topically and/or systemically) to the suspected onychomycosis.

### Routine OM laboratory testing

For routine OM laboratory testing, samples were prepared for microscopic/histologic examination and culture. Material from nail scrapings was taken from petri dishes and wet mounts were created by combining the material with one drop of Blankophor dye (Indulor, Ankum, Germany) on a glass slide for fluorescence microscopic examination. The remaining material was then implanted in Dermatophyte-Agar with or without the mold inhibitor Cycloheximid (Merck, Darmstadt, Germany). The plates were then incubated for three to four weeks at room temperature with weekly assessment by an experienced dermatologist.

### Molecular OM laboratory testing

DNA was extracted using the QIAmp DNA mini kit (Qiagen, Hilden, Germany) following the manufacturer’s instructions for tissue samples. 40 ml of Proteinase K (Qiagen, Hilden, Germany) were added prior to the incubation at 56 °C and 1000 rpm overnight. Half of the sample volume was further used for sequencing preparation. From the remaining quantity, multiplex PCR followed by incubation on a chip for detection of specific fungal sequences were performed using the EUROArray Dermatomycosis kit (EUROIMMUN, Lübeck, Germany) according to the manufacturer’s instructions. The PCR settings prior to hybridization onto the microarray chip were as follows: initial denaturation for 3 min at 94 °C, 36 cycles of each 15 s at 94 °C, 15 s at 56 °C and 40 s at 72 °C, followed by 1 min final elongation at 72 °C.

### Library preparation and NGS

From the isolated DNA samples, the nuclear ribosomal ITS2 region was amplified by using a dual indexing approach and the ITS specific sequencing primers ITS4 and fITS7 that were linked to a unique eight-base multiplex identifier, as previously described^8^. PCR reactions were carried out in a 25 µl reaction volume using the Phusion^®^ Polymerase (ThermoFisher, Waltham, MA, USA) with the following PCR settings: initial denaturation for 30 s at 98 °C; 35 cycles of 9 s at 98 °C, 30 s at 50 °C, and 30 s at 72 °C, final extension for 10 min at 72 °C. For all forward and reverse primer combinations, template-free reactions were performed. PCR products were quantified on a 1.5% agarose gel (Biozym, Hessisch-Oldendorf, Germany) after 5 min at 120 V followed by 1 h at 110 V. Gel pictures were taken on a Vilber E-Box CX5.TS (Vilber Lourmat SAS, Collegién, France) and image analysis was done using the image analysis software Vision quant version 16.16.0.0 (Vilber Lourmat SAS, Collegién, France). Fungal PCR products were equimolarly subpooled, run on an UltraPure™ Agarose gel (ThermoFisher, Waltham, MA, USA) and the 250 bp sized bands were extracted from the gel using the MinElute Gel Extraction Kit (Qiagen, Venlo, Netherlands). Subpool concentrations were determined using the NEBNext Library quantification Kit for Illumina (New England Biolabs, Frankfurt am Main, Germany) on the Eppendorf Mastercycler ep Realplex (Eppendorf, Hamburg, Germany) following the manufacturer’s instructions. The subpools were combined to an equimolar library which was further purified using the AMPure^®^ Beads XP Kit (Beckman&Coulter, Brea, CA, USA) and quantified with the NEBNext Library quantification Kit. The average amplicon size of the library was determined using the Agilent High Sensitivity DNA Kit (Agilent, Santa Clara, CA, USA) and the library (17.5 pM) was sequenced on a MiSeq (Illumina, San Diego, CA, USA) using the MiSeq v3 600 cycles sequencing chemistry (Illumina, San Diego, CA, USA) together with 20% PhiX Control v3 (Illumina, San Diego, CA, USA). Sequencing data used for this study were submitted to the European Nucleotide Archive (ENA) and is available under accession number PRJEB37496.

### Bioinformatics analysis

Raw FASTQ files that were obtained from the MiSeq sequencing platform included about 18.8 million reads. Raw data was processed using PIPITS^8^ (v2.3) with default parameters and the RDP Classifier^14^ (v2.1211) against UNITE database^15^ (04.02.2020) to retrieve taxonomic annotation for each sequence. Statistical analysis was performed using R^16^ (v4.0.2). The R packages *phyloseq*^17^ (v1.28), *vegan*^18^ (v2.5.6), *PhILR*^19^ (v1.14.0), *DivNet*^20^ (v0.3.6), *caret*^21^ (v6.0-86) and *indicspecies*^22^ (v1.7.6) were used to facilitate the subsequent analyses. Visualization was done with *ggplot2*^23^ (v3.2.1) and *ggpubr*^24^ (v0.2.3). Post-processing was facilitated as follows: Filtering of the data set comprised removal of unassigned phyla, removal of OTUs prevalent in less than five percent of samples and removing samples with less than 1000 counts in total. The filtered data set was comprised of 59 samples (40 cases and 19 controls) containing 338 OTUs. The lack of exhaustively curated databases for fungal genomes resulted in a growing number of unassigned entities corresponding to the taxonomic rank, e.g., on family level about one in four are unassigned while on species level more than half of the entities remain unspecified (Suppl. Fig. 1). Nevertheless, NGS followed by taxonomic annotation allows to identify fungal species. An overview of per sample communal composition, over all taxonomic ranks, is provided in Supplementary Fig. 2. Cumulative Sum Scaling (CSS) was employed as normalization method to mitigate the bias introduced by strong variability in total counts per sample (mean: 41,265; standard deviation: ± 62,937). The subsequent transformation to relative abundances enabled comparison of samples.

For linear regression analysis, lm (R *stats* package v4.0.2) was used and the Bayesian model was build using *rstanarm*^25^ (v2.21.1). Results from both models were plotted using the R package *sjPlot*^26^ (v2.8.5). The R package *randomForest*^27,28^ (4.6–14) was used to build the Random forest classifier. Genus count abundances were transformed to relative abundances and 70% of the data were used as training data set. The remaining 30% were used as validation set (held-out data). For classification, 10,000 trees were generated, and 40 variables were tested (mtry parameter). Additionally, the proximity between rows was calculated.

Sample-wise abundance plots were generated from phylum to genus level by selecting the 20 taxa with highest mean abundance and agglomerating the remaining entities into the single category called ‘others’. In the analysis we quantified the fungal diversity on the family level, as it constituted the best trade-off between degree of detail and mapping quality. Group-wise tests for difference in taxonomic abundance were performed using Mann–Whitney *U* test and *p* values were corrected for multiple testing using Benjamini–Hochberg correction. Alpha diversity (Shannon diversity) on non-normalized counts was estimated using *DivNet* (sample-wise and community-wise) according to the assumption that taxa in the community cooccur in an ecological network^29^. Differences in alpha diversity were assessed using the betta function (*breakaway* v4.7.2) for sample-wise estimates with group as a fixed factor and testHypothesis (*DivNet*) for community-wise estimates, respectively. Beta diversity was estimated by first performing a phylogenetic isometric log-ratio transformation on the filtered phyloseq object. To that end, the filtered (non-normalized or scaled) compositional data set was transformed via the *PhILR* package, which facilitates the estimation of diversity in terms of Euclidean distance measure. Subsequently, a two-way permutational multivariate analysis of variance (PERMANOVA) testing (99,999 permutations) was conducted on the resulting distance matrix in order to evaluate the contribution of the covariates “Therapy”, “Pets”, “Sex” and “Age” as well as the difference between controls and cases. Whereby “Therapy” indicates if any treatment was applied for suspected OM, and “Pets” indicates if the person has regular contact with pets or domestic animals. The significance of testing was controlled by analyzing the respective dispersion of beta diversity, i.e., evaluating if compared groups are homogenously distributed. The association between species and membership to a site-group was calculated using the signassoc function as implemented in the *indicspecies* package.

### Ethics approval

Ethics Committee of the University of Lübeck, AZ: 17-066.

## Results

A total of 284 participants were enrolled in this study, the majority (n = 249) presented to our in- and out-patient clinic with suspected OM. Clinical presentation, “conventional” and molecular diagnostics were used to classify the participants as either healthy controls or confirmed OM cases. Samples were categorized as healthy, if clinical examination led to no suspicions of OM and none of the diagnostic tests was positive for OM, and as cases if OM was clinically suspected and at least one positive finding from any of the diagnostic tests applied. Overall, 44 case samples and 31 controls were included in the ITS sequencing procedure. From the total of 75 sequenced samples, 59 samples (40 cases, 19 controls) passed the quality control of the PIPITS analysis pipeline. They contained more than 1000 contigs respectively and comprised a total of 338 OTUs.

### Composition of toenail fungal communities identifies distinct subgroups in the case samples

**Figure 2:**
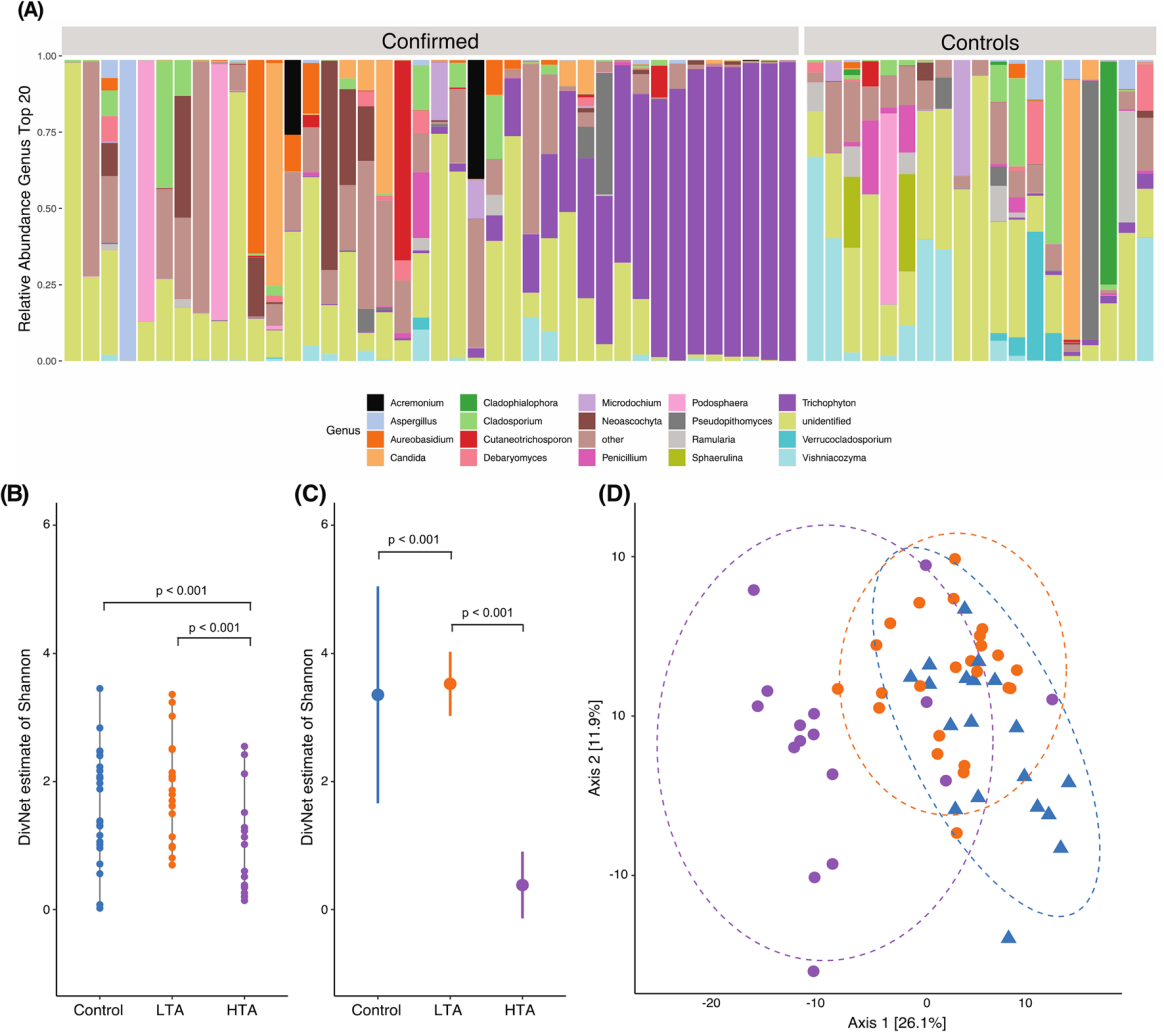
Composition of toenail fungal communities. (**A**) The 20 most abundant fungal genera (average across samples) were selected for visualization The category others comprises all remaining genera Samples are depicted in cohort grouping of confirmed case- and healthy control samples Within those groups the samples were arranged based on the abundance of genus *Trichophyton* in order to visualize the found subdivision in the case group. (**B**) Alpha diversity in sample-wise estimates showed no difference between controls and LTA group Compared to the control group a significant decrease in Shannon diversity was observed (*p* < 0.001, decrease 0.8934, s.e. 0.1886) in HTA samples. (**C**) Estimated alpha diversity in community-wise comparison using Shannon The low *Trichophyton* abundance (LTA) group showed a significant increase in diversity compared to the healthy controls (*p* = 0.001, increase 0.1719, s.e. 0.0511) The high *Trichophyton* abundance (HTA) group had a significantly lower diversity compare to the control group (*p* < 0.001, increase 2.9728, s.e. 0.0652). (**D**) Principle coordinate analysis (PCoA) of case/control samples based on the Euclidian distance on PhILR transformed counts with blue triangles referring to controls, orange circles to LTA cases and violet circles to HTA cases Axis 1 capture 26.1% of the variation and separates the cases into two distinct groups.

In healthy controls, we observed a large variation in the abundance of different fungal communities. For example, on the family level, in 5/19 samples the family *Bulleribasdiaceae* was most abundant, accounting for 35% or more of all reads (Suppl. Fig. 1). In addition, 4/19 samples were dominated by *Mycospharellaceae*, whilst *Cladosporiaceae* was most abundant in 3 samples. The remaining 7 samples did not have a common dominating family (Suppl. Fig. 1). This variation in healthy controls was even more apparent on the genus level, where most compositions of the toenail mycobiome are unique to the individual (Fig. 2A). This was confirmed by the large variance in alpha diversity (Fig. 2B).

In patient samples, we noted a remarkable separation of the mycobiome family and genus level into two distinct groups (Suppl. Fig. 1, Fig. 2A). In 16/40 OM cases, the family level was dominated by *Arthrodermataceae*, which were almost exclusively of the *Trichophyton* genus, accounting for at least 80% of the reads in various instances. In the remaining 24 OM patient samples, fungal diversity was very similar to that of healthy controls. Thus, the OM patient group can be stratified according to the presence of the genus *Trichophyton*: Those with a low *Trichophyton* abundance (below 15%), and those with high *Trichophyton* abundance (above 19%, Fig. 2A, Suppl. Fig. 3A).

### OM samples can be classified into two distinct subgroups

According to this finding, samples were stratified into healthy controls, low *Trichophyton* abundance (LTA) cases or high *Trichophyton* abundance (HTA) cases, for subsequent analyses (Fig. 2B–D). The robustness of this grouping was assessed using linear and a Bayesian regression model alongside the assigned group as explanatory variable and relative abundance of *Trichophyton* as response. Both models confirmed the marked increase in *Trichophyton* abundance in the high group (linear model: R^2^_adj_ = 0.753, *p* < 0.001; Bayesian model R^2^ = 0.748, 95% CI 0.55–0.80; Suppl. Fig. 3C–D). We further classified the samples by a Random forest approach, which makes use of the relative abundance of all identified genera. The trained model had no out-of-bag error and was able to stratify the held-out data correctly into groups with low and high *Trichophyton* abundance. The genus with the highest overall impact on the stratification was *Trichophyton* (mean decrease in Gini index of 5.2; Sup. Fig. 3E).

We subsequently determined the fungal species richness and their heterogeneity using the alpha diversity of the fungal communities. Differences in sample-wise estimates of diversity were modelled to test for differences of total diversity (observed plus unobserved) across multiple samples^30^. A significant decrease in diversity was detected between healthy controls and cases (betta test, *p* < 0.001, decrease 0.5204, s.e. 0.1350). However, no differences in alpha diversity were observed between healthy controls and LTA samples (betta test, *p* = 0.105). Furthermore, the HTA group showed a decreased Shannon diversity compared to healthy controls (*p* < 0.001, decrease 0.8934, s.e. 0.1886) as well as compared to the LTA group (*p* = 0.002, decrease 0.6217, s.e. 0.1677, Fig. 2B). Besides estimating alpha diversity for each sample, Shannon index was estimated for each group (community-wise) as well (Fig. 2C) and further investigated applying a model that accounts for both strong heterogeneity between samples and rare taxa^30^. As in the sample-wise data, Shannon diversity was significantly decreased in cases compared to controls (*p* < 0.001, decrease 0.7137, s.e. 0.0882). The LTA group showed a significant increase in the community richness compared to the healthy controls (*p* = 0.001, increase 0.1719, s.e. 0.0511). Furthermore, the HTA group had a significantly lower diversity compared to the healthy controls (*p* < 0.001, increase 2.9728, s.e. 0.0652, Fig. 2B–C).

Next, beta diversity, i.e., the variation in mycobial composition between groups, was investigated and showed a significant difference (*p* = 4 * 10^−5^, R^2^ = 0.08442) between cases and controls, accounting for approximately 8% of variation in the data set. Examination of group dispersions showed significant differences in the dispersion of case and control samples. This is congruent with the separation of cases into LTA and HTA samples, as it indicates that the difference between case and control group is influenced to a large degree by the difference of composition within groups. After accounting for the subdivision into LTA and HTA groups we found that the differences are significant (*p* = 1 * 10^−5^, R^2^ = 0.18055) and account for 18% of variation in the data set. The covariates age, sex, presence of pets and previous antifungal therapy were not found to be significant and account in total for 14.5% of variation in the data set. Validation by testing for differences in beta dispersion showed no significant difference in composition within the three groups, i.e., the results of the multivariate analysis are not an artifact of heterogenous dispersions (Fig. 2D).

### Fungal sequencing does not significantly outperform existing diagnostics

Lastly, we addressed the question whether sequencing-based diagnosis outperforms current diagnostic approaches, including PCR-based methods. To that end, a supervised Random forest approach estimated the efficacy of separating cases from controls based on patient data, i.e., covariates, PCR results and OTUs. We considered the division into control, LTA and HTA groups as ground truth and estimated the classifier performance in a bootstrapped training and test approach. A detailed description of the procedure as well as receiver operator curve (ROC) statistics are shown in Supplementary Fig. 4. In fact, the classification based on OTUs marginally, but not significantly, outperformed the other features with an average probability of 91.27% of correct classification. However, as expected based on the strong similarity in composition between control and LTA groups, the later could not be identified reliably.

## Discussion

We used current diagnostic tools of OM to curate a cohort of healthy and OM samples. The utilization of NGS enabled us to investigate the complex composition of the mycobiome in healthy and infected toenails. Based on previous reports, we had expected a predominance of genus *Malassezia* in healthy samples. We further hypothesized that by occupation of the toenail niche with *Malassezia* species protects from onychomycosis—albeit some *Malassezia species* may cause OM^31^. The obtained results were, however, quite unexpected in several ways:

In our study, only those nail samples were included, in which clinical examination, microscopy, histology and multiplex analysis excluded OM. In these samples, a much greater diversity was observed and none of the samples was dominated by *Malassezia*. In the study by Findley and colleagues the predominant species were different *Malassezia* species^12^. By contrast, we herein report a much higher diversity on both genus and species level, in toenails from healthy controls. At the moment, we can only speculate on the reason for this difference. We found the ongoing effort to curate more fungal genomes which is leading to a lack of comprehensive databases to map against, to be the likeliest explanation. Currently, this leads to decreasing accuracy in assignment on genus and species level, up to an amount of 53% unassigned reads on species level. Other, mutually non-exclusive explanations, are: (i) a high plasticity of the mycobiome, as described for the intestinal mycobiome (ii) relative low sample sizes of both studies, (iii) different selection criteria, (iv) regional, and/or (v) seasonal differences.

At an about equal distribution, the case group was stratified by the abundance of the genus *Trichophyton*. Within this cohort, we found an abundance of 15% *Trichophyton* to be the boundary that separates the high *Trichophyton* abundance (HTA) samples from the low *Trichophyton* abundance (LTA) samples. This finding suggests that OM can be either pathogen- or host-/environment-driven. In accordance with this, we showed that on average, the sample-wise calculated fungal alpha diversity was significantly lower (*p* < 0.001) in HTA samples compared to healthy controls, which is an expected confirmation of our alternative stratification in the case group. No significant differences were found between controls and LTA group. However, considering each group as a single environment, i.e., compute group-wise alpha diversity, results in significantly more diversity in the LTA group as compared to healthy controls. This means that dysregulated communities, associated with disorders, do not necessarily have to be less diverse than healthy communities. Furthermore, the state of dysregulation as a whole is more diverse than the stable healthy state. One conceivable explanation is that the LTA samples are a snapshot of the onset of OM while the HTA group represents late-stage disease, i.e., during the state of dysregulation a single pathogen found favorable conditions to become the dominant entity. This is congruent with the growing severity of symptoms during disease progression.

Due to the similarity in fungal beta diversity between a subclass of OM patients with healthy controls, ITS2 sequencing, followed by taxonomic annotation, might currently not be suited for the diagnosis of OM. Thus, diagnosis of OM still seems to be a domain of conventional microscopy and culture, which may be partially replaced by sequencing-based multiplex assays^6,32^. However, ITS sequencing, in addition to conventional/molecular OM diagnosis, may provide significant clues regarding the underlying mechanics of infection and disease progression, i.e., whether or not additional host- and/or environmental factors need to be considered for treatment and prevention. Current treatment of OM predominantly targets the causative fungal pathogen, while considering host- and environmental factors, e.g. age, presence of diabetes or occlusive footwear, as risk factors for an unsatisfactory treatment response and recurrence^33^. A more comprehensive understanding of disease progression and interaction with host- and environmental factors could potentially offer new targeted approaches for individual treatment. In this context the LTA group is of particular interest as the relationship between mycobial composition disease status is more ambiguous than in the HTA group.

The distribution of fungi in individual patients points towards potentially clinically relevant and interesting findings. More specifically, we observed a relative high abundance of *Neoascochyta graminicol*a in 5/24 OM patients of the LTA group. By contrast, this species was not detected among the 20 most abundant species in the HTA or the healthy control group (Supplementary Fig. 1). *Neoascochyta* are commonly found on plants^34^ and have not been associated with human disease so far. In addition, two OM patients in the LTA group show a predominance of *Podosphaera* (Fig. 2). This genus of fungi causes powdery mildew in plants^35^. Up to date, there is no report on *Podosphaera* as the causative agent of OM or tinea. Hence, the presence of *Podosphaera* is most likely due to colonization rather than disease causing-agent. Furthermore, one OM patient in the LTA group had a high presence of the species *Schizopora paradoxa* (*Xylodon paradoxus*), which is a rot wood degrader commonly found in Korea^36^. Again, this species has so far not been associated with human pathology. Thus, the detection of *S. paradoxa* most likely is most likely due to colonization. However, larger cohorts of OM patients are needed to confirm or reject these assumptions.

Our study is not without limitations: First, the age distribution among the groups is different. Healthy individuals are on average 20 years younger than the diseased patients. This may have an impact on the mycobiome as skin physiology changes with age^37^. But this is, in accordance with the relevant literature, as it points out that OM predominantly affects the elderly^2–4^. However, the setup of the experiments aimed at identification of OM patients confirmed by at least one diagnostic method and excluding clinically normally appearing controls where fungi were detected by any of the current diagnostic methods. We believe the later to be of more importance for this exploratory study. The impact of age on the fungal diversity would be an interesting follow-up study.

Second, the reported higher prevalence of OM in men^2,3^ is interesting with respect to the HTA and LTA group separation. We found a significant difference in distribution of the sexes between controls and HTA group. While the distribution of sexes in the LTA group is equal, the HTA is predominantly composed of male subjects. The existing bias towards males in the case group could be a result of the enhanced health-care behavior of women compared to that of men. It has been documented that men tend to delay seeking medical advice until a disorder becomes a severe inconvenience^38,39^. Thus, we hypothesize that the higher abundance of Trichophyton rubrum is a phenomenon observed in infections at a later stage.

Collectively, we here provide new insights into the human nail mycobiome in health and OM. In addition to the detailed insights on fugal diversity, our results also indicate that OM develops either due to the high pathogenicity of certain fungal pathogens, or, alternatively, by yet-to-be-defined host or environmental factors.

## Supplementary Information

Supplementary Information.

## Data Availability

Sequencing data used for this study were submitted to the European Nucleotide Archive (ENA) and is available under accession number PRJEB37496.
