# Supplementary material for: Biodiversity of mycobial communities in health and onychomycosis
Source: Sci Rep. 2022 May 25;12:8872. doi: 10.1038/s41598-022-13074-8 (PMC9133011; doi:10.1038/s41598-022-13074-8)
Supplement: Supplementary file 1 — Supplementary Information. [file 41598_2022_13074_MOESM1_ESM.pdf]

Biodiversity of mycobial communities in health and onychomycosis

Michael Olbrich, Anna Lara Ernst, Foteini Beltsiou, Katja Bieber, Sascha Ständer, Melanie Harder, Waltraud Anemüller, Birgit Köhler, Detlef Zillikens, Hauke Busch, Axel Künstner, Ralf J Ludwig

Corresponding author. Ralf J. Ludwig, Institute for Experimental Dermatology, University of Lübeck, Ratzeburger Allee 160, 23562 Lübeck, Germany, phone: +49 (0) 451-500-41686, email: ralf.ludwig@uksh.de, ORCID: 0000-0002-1394-1737

Corresponding author. Hauke Busch, Lübeck Institute for Experimental Dermatology; Institute for Cardiogenetics, University of Lübeck, Ratzeburger Allee 160, 23562 Lübeck, Germany, phone: +49 (0) 451-3101-8470, email: hauke.busch@uni-luebeck.de, ORCID: 0000-0003-4763-4521

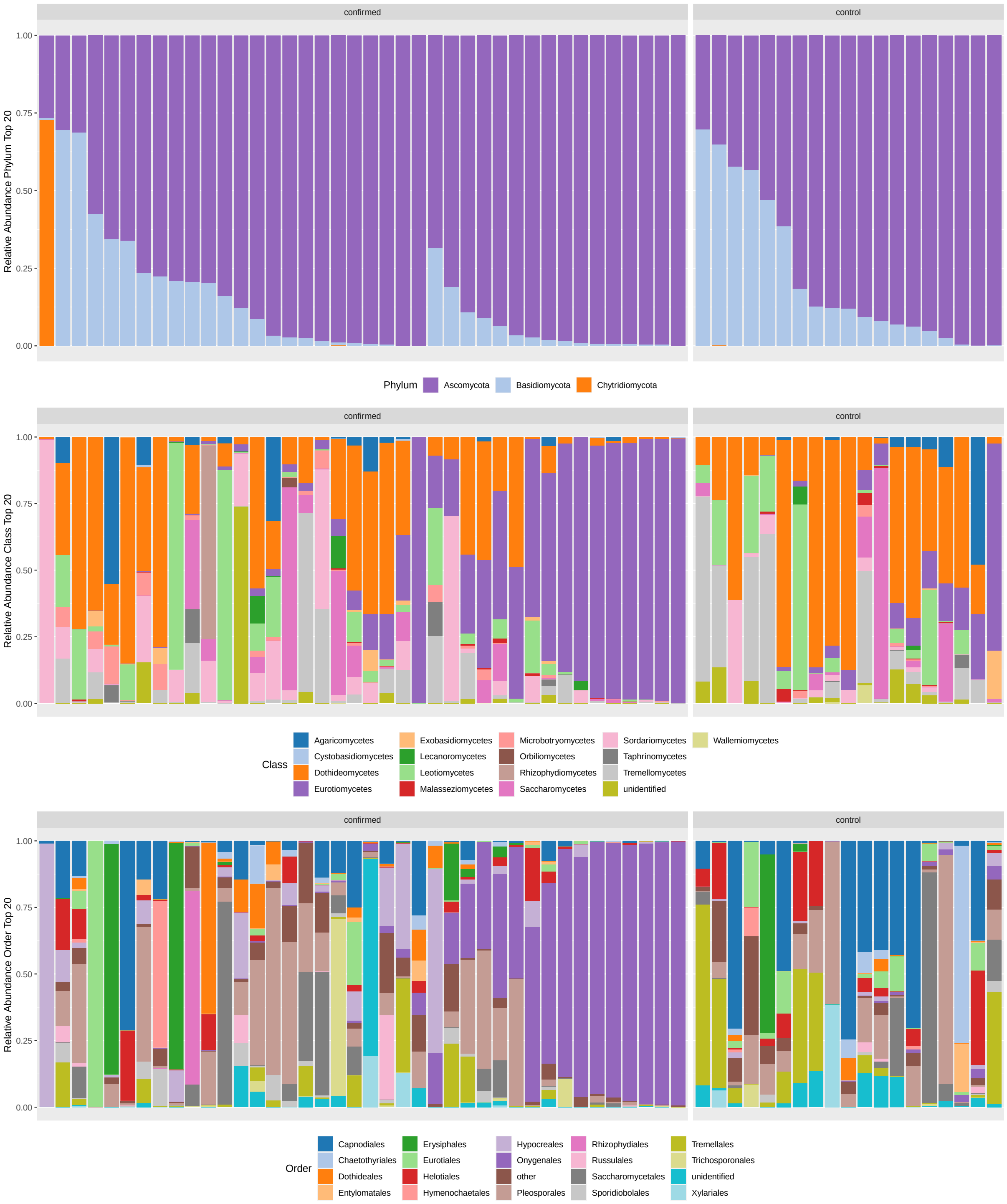

confirmed
control
Relative Abundance Phylum Top 20
1.00
0.75
0.50
0.25
0.00
Phylum
Ascomycota
Basidiomycota
Chytridiomycota
Relative Abundance Class Top 20
Class
Agaricomycetes
Cystobasidiomycetes
Dothideomycetes
Eurotiomycetes
Exobasidiomycetes
Lecanoromycetes
Leotiomycetes
Malasseziomycetes
Microbotryomycetes
Orbiliomycetes
Rhizophydiomycetes
Saccharomycetes
Sordariomycetes
Taphrinomycetes
Tremellomycetes
unidentified
Wallemiomycetes
Relative Abundance Order Top 20
Order
Capnodiales
Chaetothyriales
Dothideales
Entylomatales
Erysiphales
Eurotiales
Helotiales
Hymenochaetales
Hypocreales
Onygenales
other
Pleosporales
Rhizophydiales
Russulales
Saccharomycetales
Sporidiobolales
Tremellales
Trichosporonales
unidentified
Xylariales

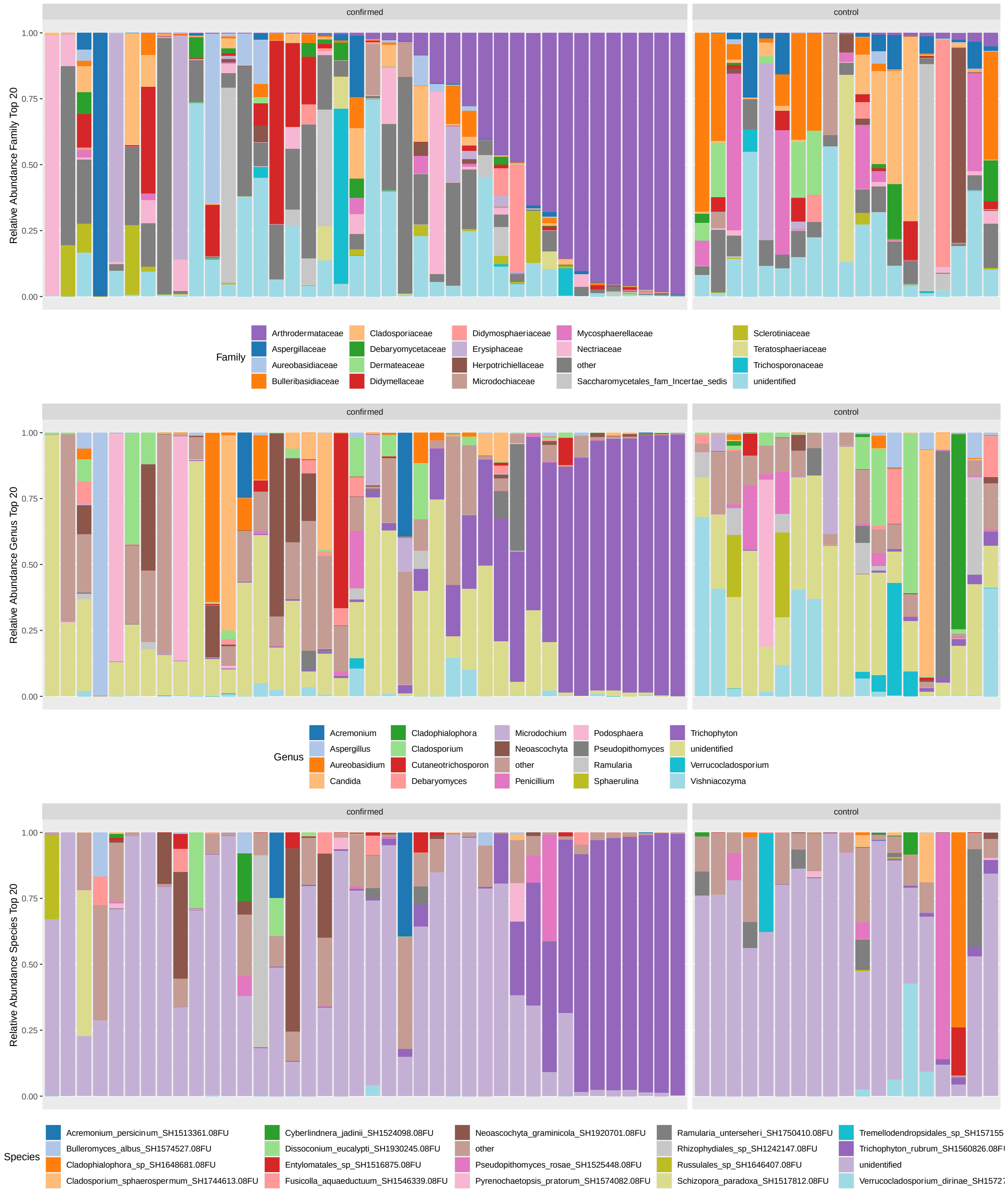

Supplementary Fig. 1 Composition of the top 20 most abundant fungal communities in toenail scrapings on taxonomic levels Phylum to Species. Samples are depicted in cohort grouping of confirmed case- and healthy control samples. Within those groups the samples were arranged based on the abundance of genus Trichophyton in order to visualize the found subdivision in the case group.

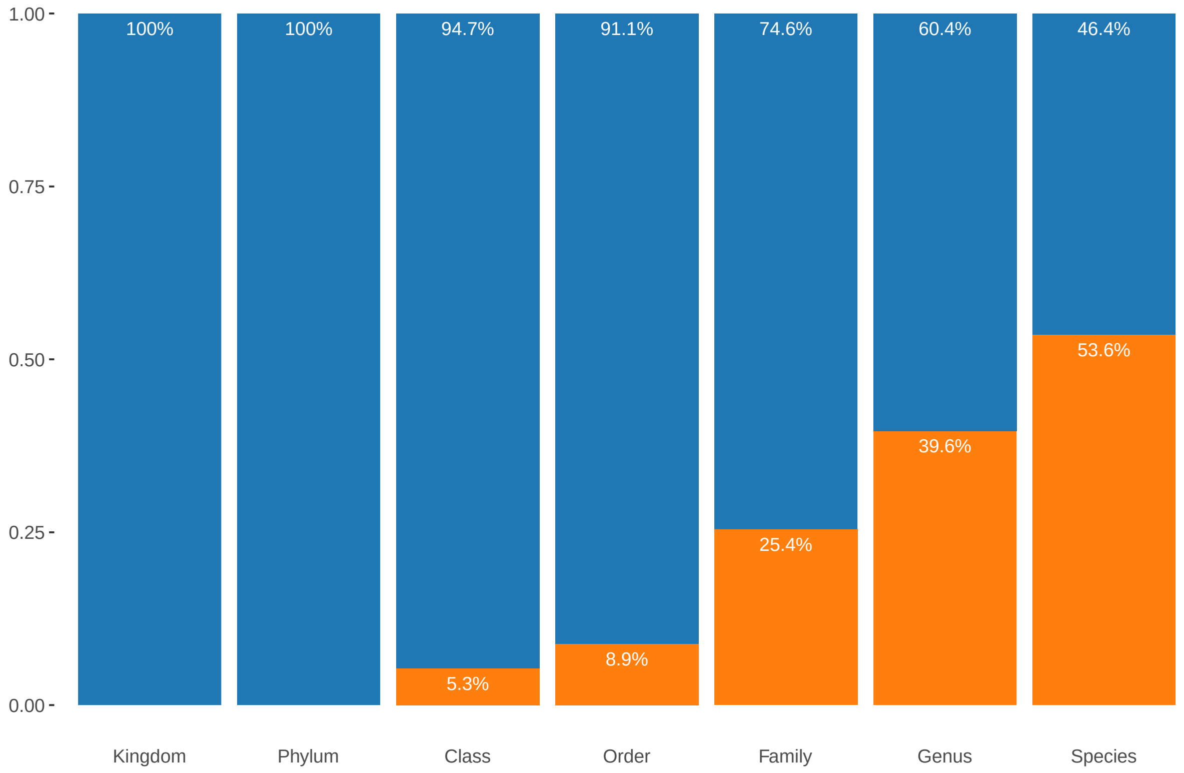

Supplementary Fig. 2 Proportion of classified (blue) and unclassified (orange) entities for each taxonomic level. Below Phylum level, the number of unmapped reads increases strongly. This results in a blurring of communal composition with taxonomic level, e.g., more than half of the species could not be assigned.

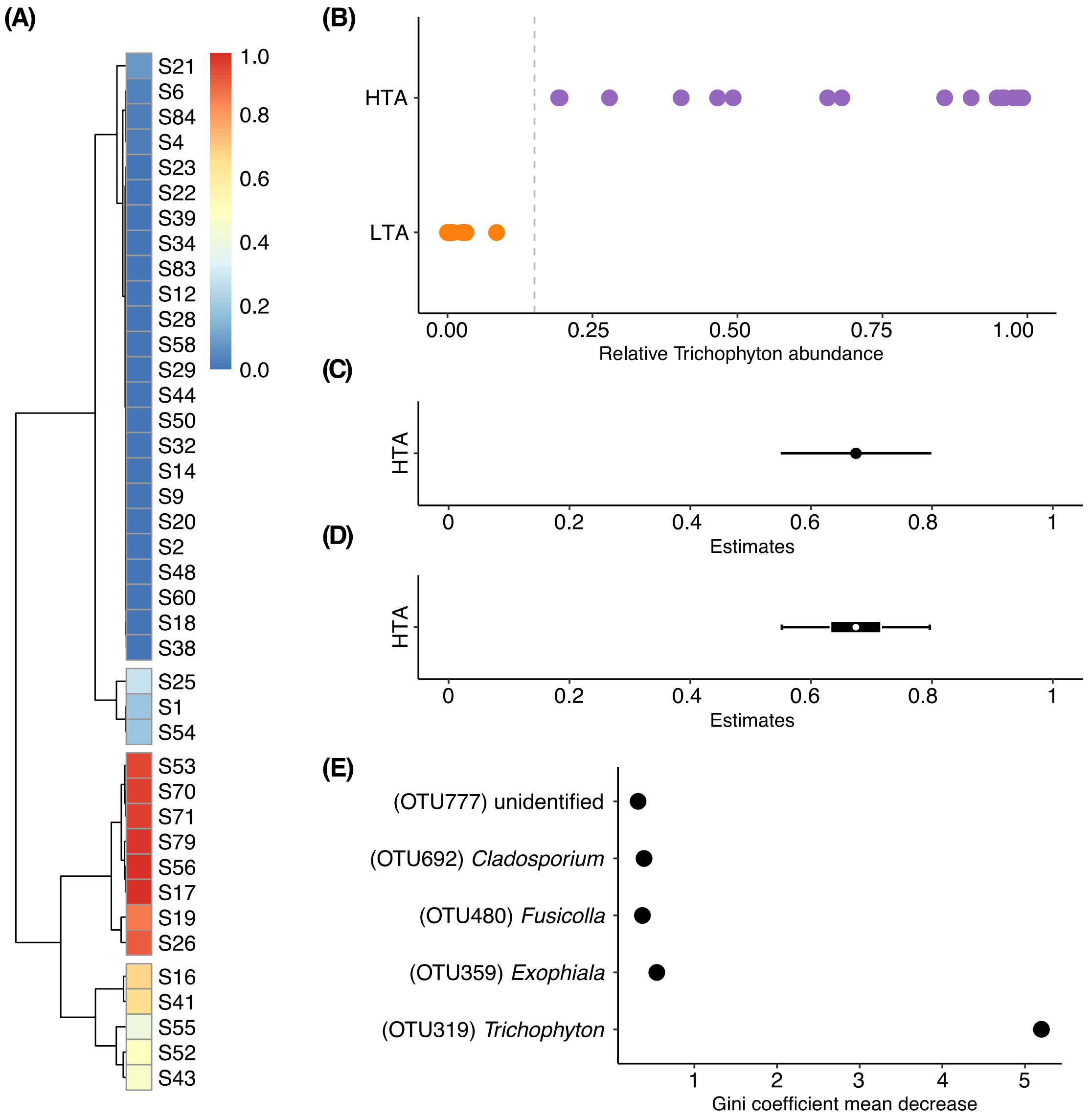

Supplementary Fig. 3 Identification of the genus Trichophyton as main driver for differences in the disease group. The heatmap in panel **(A)** shows the relative abundance of Trichophyton across all confirmed onychomycosis cases (blue refers to no/low Trichophyton and red to high abundance; intermediate abundance is colored by yellow). The optimal number of cuts in the tree was estimated using gap-statistics (1,000 bootstraps, maximum number of clusters set to 20). Panel **(B)** shows the relative abundance of Trichophyton after stratifying the cases into groups of low (orange) and high (violet) Trichophyton abundance. The dashed line indicates 15% of Trichophyton abundance. The model estimates for the HTA group are shown for the linear model **(C)** and the Bayesian model approach **(D)** whiskers denote the 95% interval of outer probabilities for the uncertainty intervals and the box the 50% interval of inner probabilities. **(E)** The mean decreases in Gini coefficient for all genera with a mean decrease above 0.3 from the Random forest classifier.

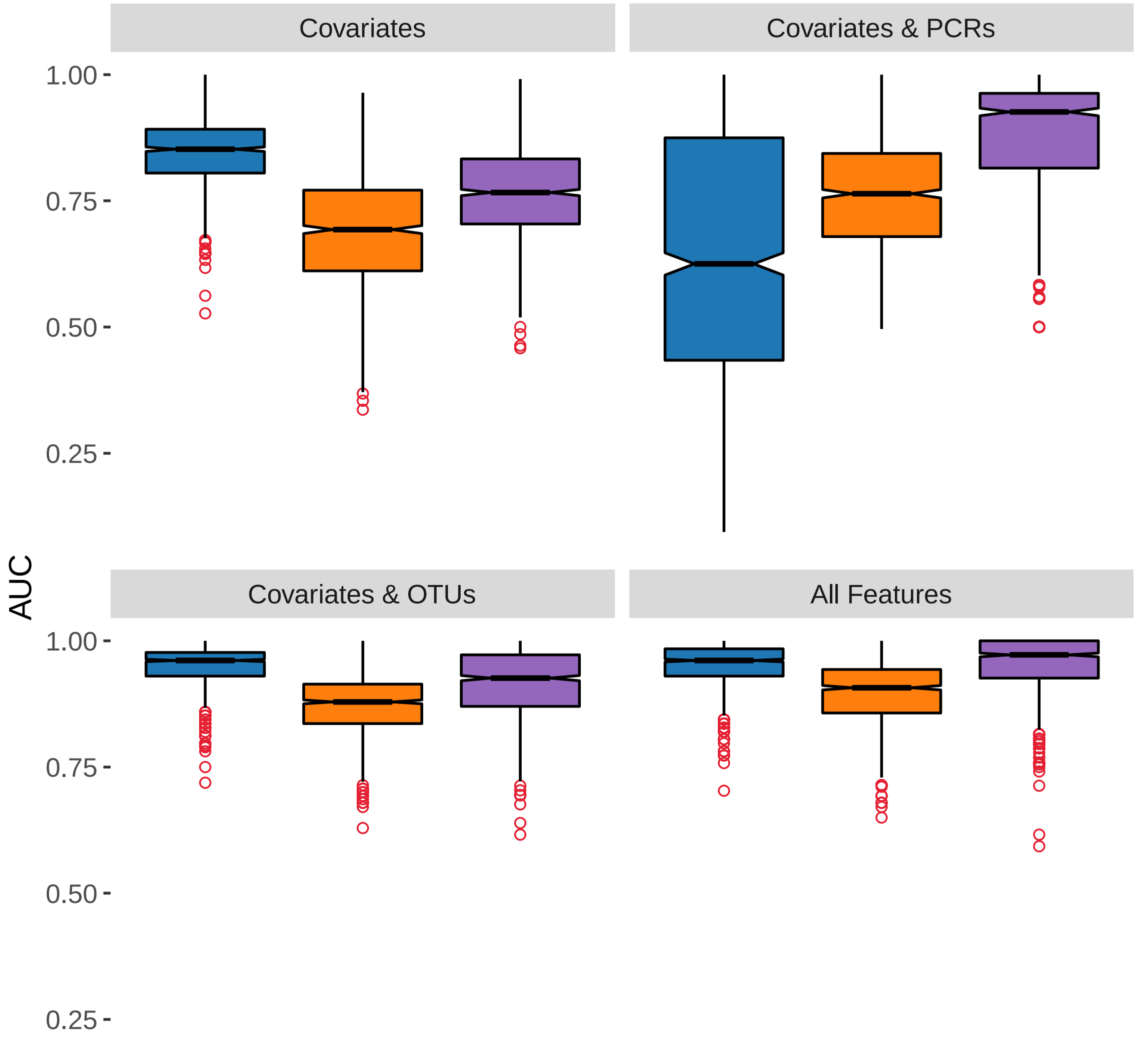

Supplementary Fig. 4 ITS2 sequencing as a diagnostic tool evaluated by the performance of a Random forest classifier in distinguishing the three distinct subsets in the cohort. The following groups were identified: healthy controls (blue), LTA case group (orange) and the HTA sub-group of cases (purple). We employed a training and test procedure to evaluate the classifiers performance in a bootstrap approach, based on reclassification on 1,000 randomly selected training sets with proportion of 70/30 training to test set size. The input-features are comprised of covariate information (Sex, Pets, Age), PCR-based diagnostics and the OTU abundances. We estimated the mean and standard deviation of the classifiers' performances, which were quantified via the area under the curve (AUC) statistics. To that end we computed the receiver operator curve (ROC), i.e., true positive rate in relation to false positive rate, and calculated the area under the curve (AUC). The AUC can take values in the interval I= [0,1] indicating the probability of correct classification, i.e., an AUC of 0.5 signifies a 50% chance of correct classification. Thus, a biomarker for medical use would require a value close to 1. The classification based on OTUs outperformed the other features with an average probability of 91.27% of correct classification.
